# Successful management of pregnancy in Turner syndrome (Monosomy X): A rare condition-based learning experience from Vietnam

**DOI:** 10.18502/ijrm.v22i5.16442

**Published:** 2024-07-08

**Authors:** Ngoc Bich Trinh, Anh Dinh Bao Vuong, Phuc Nhon Nguyen

**Affiliations:** ^1^Department of High-Risk Pregnancy, Tu Du Hospital, Ho Chi Minh City, Vietnam.; ^2^Tu Du Clinical Research Unit (TD-CRU), Tu Du Hospital, Ho Chi Minh City, Vietnam.

**Keywords:** Cesarean section, Materno-fetal outcome, Oocyte donation, In vitro fertilization, Turner syndrome.

## Abstract

**Background:**

Turner syndrome (TS) is recognized with partial or complete loss of the second sex chromosome, occurring in approximately one in 2500 live births, and related to high failure of pregnancy. However, along with the advantage of assisted reproductive technology, the cases of TS pregnant women have been recently addressed worldwide. Therefore, the reproductive health of TS pregnant women should be a concern by physicians and obstetricians, particularly, in the low-middle income countries with low-resource settings.

**Case Presentation:**

Here, we describe a rare case of term pregnancy on a TS woman (45, XO) receiving oocyte donation at a private fertility center. Later, the woman was monitored uneventfully during antenatal care and hospitalized at our center for a cesarean delivery with favorable pregnancy outcomes at term.

**Conclusion:**

To our knowledge, this is the first report relating to a particular pathology in Vietnam. Through this case report, we would like to emphasize the novel opportunity for TS women desiring parents, thus raising an appropriate awareness of healthcare providers.

## 1. Introduction

Turner syndrome (TS) is one of the most frequent chromosomal abnormalities affecting females, with a prevalence estimated to be 1 of 2500 live births (1). For most individuals with TS, the diagnosis is identified during the adolescence stage due to a lack of pubertal development (2). TS is potentially related to infertility because of primary ovarian insufficiency. The TS can carry a pregnancy at the rate of 8% and this increased in mosaic detection of TS (3). The role of in vitro fertilization with oocyte donation is an appropriate option choice for TS women who desire parenting (4, 5).

However, pregnancy in women with TS is known to be at high risk because of spontaneous miscarriages and potential cardiovascular complications which can be life-threatening and reduce the survival rate (6, 7). The large metacentric study relating to oocyte donation in TS in 10 French oocyte donation centers between 2012 and 2016, included 73 pregnant women with 39 pregnancies, of which only 24 children were born healthy. On the other hand, almost all cases were associated with adverse materno-fetal outcomes (1).

Therefore, all these women should be necessarily screened with comprehensive cardiovascular counseling before pregnancy and have a proper follow-up during pregnancy as well as postpartum.

Herein, we present a rarely success case of 45, X TS pregnant women at our tertiary center, thus raising awareness among healthcare providers.

## 2. Case Presentation

**Figure 1:**
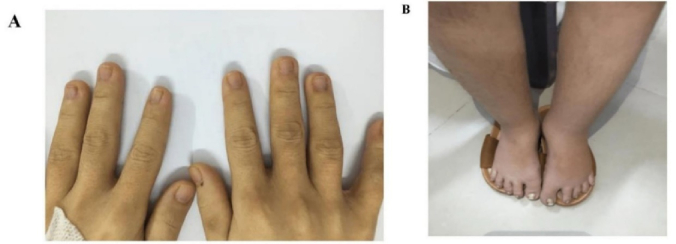
A) Short fingers, B) Short legs, edema in the foot, and hirsutism.

A 30-yr-old pregnant woman was hospitalized due to edema in her foot for 1 wk (Figure 1 A-B). The woman was diagnosed with TS on karyotype with 45, X monosomy when she was 15 yr old with short stature, amenorrhea, and no signs of puberty because of premature ovarian failure. Initially, she was treated with hormonal replacement therapy. Her medical record was also noted with congenital hypothyroidism, hydronephrosis, cholelithiasis ( > 3 mm diameter), and bilateral kidney stone ( > 8 mm diameter). She had no gestational diabetes. The protein excretion was normal. Due to the extremely low ovarian reserve (unmeasured anti-Mullerian hormone serum level), her pregnancy was conceived by in vitro fertilization with oocyte donation and preimplantation genetic testing for aneuploidy (PGT-A). In addition, the woman was diagnosed with gestational diabetes.

At admission, the vital signs were unremarkable, the woman's height was 140 cm, and her weight was 44 kg. The woman increased to 6 kg during pregnancy. However, due to the intervention of cosmetic plastic surgery, her face was less likely to have TS syndrome. On obstetric examination, the uterus was measured at 28 cm in height and the cervix was closed without vaginal bleeding. The ultrasound finding in the first trimester showed normal nuchal translucency with a normal non-invasive prenatal test. The 4-dimension ultrasound demonstrated the fetal morphologic features as normal. The current ultrasound result revealed a viable fetus, small for gestational age, weighing 2690 gr, and corresponding to 38 wk of gestation. The amniotic fluid index was estimated at 23 cm. The current ultrasound measured a biparietal diameter of 87 mm, head circumference of 327 mm, abdominal circumference of 316 mm, and femur length of 67 mm, which corresponds to the percentile of 11%, 14%, 8%, and 1% following the Hadlock ultrasound measurement based on gestational age, respectively. The blood supply was normal and the fetal heart rate was normal at 146 beats/min according to the last Doppler ultrasound. The cardiotocography was classified as group 1 following the American College of Obstetricians and Gynecologists.

**Figure 2:**
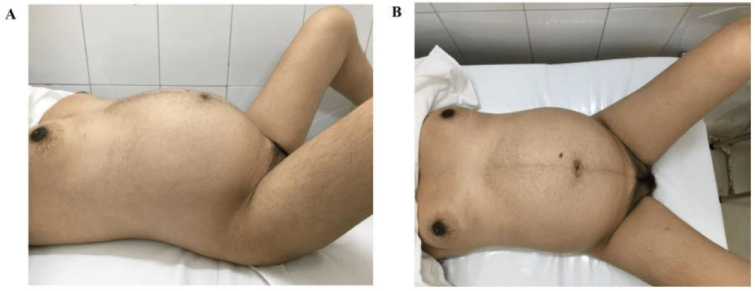
The physical body changes of TS pregnant woman with smaller breasts than that in the normal pregnant woman and hirsutism condition on the lateral side (A) and the opposite side (B).

**Figure 3:**
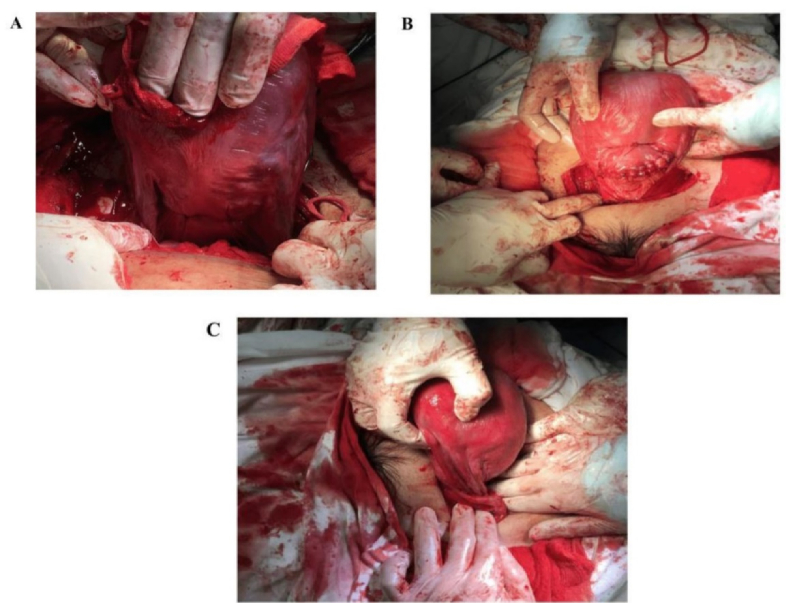
Intraoperative photos show bilateral uterine ligation (A), B-Lynch transverse compression suture and uterine closure (B), and the small uterus without the bilateral ovarian tissue (C) Compared to the normal pregnancy.

**Figure 4:**
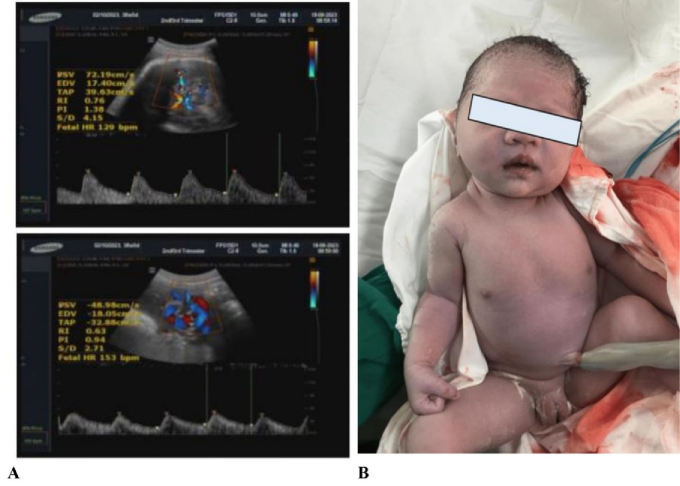
Doppler ultrasound was normally detected before cesarean section (A). A healthy newborn baby was delivered from a TS pregnant woman receiving oocyte donation (B).

Nevertheless, because of the disproportion in size between the fetus and the maternal pelvis, the woman was indicated with elective cesarean section (CS) under regional anesthesia following lung maturation with corticosteroid therapy (intramuscular dexamethasone 6 mg × 4 in 48 hr). During surgery, the estimated blood loss was measured at 400 ml, the woman was treated with medicament such as carbetocin 100 mcg preventing the postpartum hemorrhage and underwent hemostatic procedures including placental bed suture, bilateral uterine ligation, and B-Lynch transverse compression suture. The bilateral ovaries were examined with the atrophy appearance and the uterus size was smaller than the normal gravida uterus (Figure 2). The female newborn baby was evaluated with an Apgar score of 7 points at 1 min and of 8 points at 5 min (Figure 3). The newborn birthweight was 2800 gr. However, because of increased breathing frequency, the baby was sent to the neonatological care unit for monitoring and no further intervention was required. The postpartum course was experienced uneventfully. Both mother and baby were discharged on the 5 ${\;}^{\text{th}}$ day of postpartum. Doppler ultrasound was detected as normal (Figure 4).

### Ethical considerations 

This report was naturally waived by Tu Du Hospital Institutional Ethics Committee with a verbal agreement and was in accordance with the 1964 Helsinki Declaration. Informed consent was obtained from the woman before publication of this report and the use of accompanying images.

## 3. Discussion

In the present case, the woman was present with the symptoms of TS such as short stature and premature ovarian failure. Similar to our case, there are extremely high risks for women with TS during pregnancy, including aortic pathology, hepatic disease, thyroid disease, and type 2 diabetes (8). The development of hydronephrosis or obstructive nephropathy in pregnancy might occur in up to 30–40% of pregnant women with TS (9). After marriage, the woman could not conceive spontaneously due to the low anti-Mullerian hormone. Thus, the conception was promptly achieved with oocyte donation. This alternative assessment was well reported in the recent literature (1, 10). Other modalities such as ovarian tissue cryopreservation, cryopreservation of mature oocytes, and embryos could be considered for TS women desiring biological parenting (9). To avoid elective pregnancy termination because of the high risk of embryo X chromosome aneuploidy, preimplantation genetic testing should be recommended to mosaic or pure TS cases (11, 12). In this case, the patient achieved pregnancy by oocyte donation, therefore, preimplantation genetic testing was not indicated.

In our case, the woman had a successful pregnancy without spontaneous miscarriage or preterm birth although the complications of poor pregnancy outcomes are common. The fetus was assessed as small for gestational age on ultrasound. Small for gestational age was reported in approximately 18% of neonates of TS (13). Severely, congenital abnormalities such as fetal nuchal cystic hygroma, bilateral syndactyly of the hands and feet, and fetal hydrops can occur in TS pregnancy (14, 15).

Furthermore, besides the pregnancy outcomes, the poor events relating to aorta dissection and other adverse outcomes also increased since the women with TS have connective tissue defects (16). However, the complications relating to cardiovascular issues are not increased in pregnant women without prior structural heart diseases (13). A multidisciplinary team should be counseled for the strict management of TS pregnant women including materno-fetal medicine specialists and cardiologists. According to Gravholt et al., vaginal delivery is reasonable in women with TS with an ascending aortic size index below 2.0 cm/m^2^. On the contrary, a CS is recommended or a vaginal delivery with epidural anesthesia and expedited second stage may be considered in women with TS with an ascending > 2.5cm/m^2^ (16). In the present case, the CS was indicated due to the disproportion in size between the fetus and the maternal birth canal, and the pregnant woman's desire. Regardless of CS, anesthesia for cesarean delivery can be challenging since the anatomic features and low-dose combined spinal-epidural anesthesia may be the preferred technique, but the data remains limited (17).

## 4. Conclusion

To date, TS women (45, XO) can become mothers with advanced fertility treatment receiving oocyte donation although it is still a rare condition. Alternatively, this point of view should be surrogated for the prior contraindication to pregnancy regarding the TS women. Close surveillance is potentially required in these cases with adequate consultation including reproductive endocrinologists, infertility specialists, obstetricians, and cardiologists for favorable pregnancy outcomes and preventing maternal mortality.

##  Data availability

Data supporting the findings of this study are available upon reasonable request from the corresponding author.

##  Author contributions

Ngoc Bich Trinh and Phuc Nhon Nguyen designed the study and conducted the research. Ngoc Bich Trinh and Phuc Nhon Nguyen monitored, evaluated, and analyzed the results of the study. Further, Phuc Nhon Nguyen contributed to writing, reviewing, and editing the article. Anh Dinh Bao Vuong was responsible for supervision and administrative procedures. All authors approved the final manuscript and take responsibility for the integrity of the data.

##  Conflict of Interest

The authors declare that there is no conflict of interest.
